# The proportion of C1q-high and ISG15-high monocytes in the skin of patients with Behçet disease

**DOI:** 10.3389/fphar.2023.1110741

**Published:** 2023-03-07

**Authors:** Yangtengyu Liu, Ding Bao, Meng Meng, Lixia Lu, Honglin Zhu

**Affiliations:** ^1^ Department of Rheumatology and Immunology, Xiangya Hospital, Central South University, Changsha, China; ^2^ National Clinical Research Center for Geriatric Disorders, Xiangya Hospital, Changsha, China; ^3^ Provincial Clinical Research Center for Rheumatic and Immunologic Diseases, Xiangya Hospital, Changsha, China; ^4^ Department of Pathology, Xiangya Hospital of Central South University, Changsha, China; ^5^ Department of Dermatology, Xiangya Hospital of Central South University, Changsha, China

**Keywords:** bechet’s disease, vasculitis, monocytes, C1Q+mono, ISG15+mono

## Abstract

Behçet disease (BD) is a chronic systemic vasculitis that is clinically characterized by recurrent oral ulcers, genital ulcers, uveitis, and skin lesions. Here, we conducted bulk RNA-seq of skin samples from 4 BD patients and 4 normal controls (NCs). A total of 260 differentially expressed genes (DEGs), including 99 upregulated and 161 downregulated genes, were detected in the skin lesions of BD patients compared to NCs. These DEGs were mainly enriched in the following biological processes: the activation and migration of immune cells, the release of proinflammatory factors, and the IFN-γ signaling pathway. The top upregulated DEGs were CXCL10, CXCL9, FCGR3A, GBP5, GBP4, LILRB2, ADIPOQ, PLIN1, SLC43A2, and MYO1G. Using the deconvolution method CIBERSORT, we analyzed the immune cells subtypes in the skin of BD by integrating the single cell RNA-seq data from PBMC (GSE198616) and bulk RNA-seq data of skin. There was a higher proportion of C1q+ and ISG15 + monocyte subtypes in skin of BD. IHC staining of CD14 and CD16 showed that the monocyte number increased in the skin of BD. IF staining confirmed there was a higher proportion of the C1Q + Mono and ISG15 + Mono subsets in the skin of BD patients. Moreover, we analyzed the average expression level of the top upregulated genes in immune cell types found in PBMC from BD patients and NCs. Almost all the top upregulated genes expressed in monocytes. CXCL10 was specifically expressed in ISG15 + monocyte, and GBP5, GBP4 and IFI44L were expressed more strongly in ISG15 + monocytes. LILRB2 was expressed more strongly in CD16^+^ monocytes and C1Q + monocytes. In conclusion, our study identified that the IFN-γ pathway was activated in skin of BD and the proportion of C1q+ and ISG15 + monocyte subtype increased in the skin of BD.

## 1 Introduction

Behçet disease (BD) is a chronic systemic vasculitis that is characterized by recurrent oral ulcers, genital ulcers, ocular disease, and skin lesions (Sakane et al., 1999; Alpsoy, 2016; Bettiol, Prisco, and Emmi, 2020). It was first described by the Turkish dermatologist Hulusi Behçet in 1937 (Behcet, 1937). BD is a rare disease that uniquely clusters along the ancient “Silk Road,” which includes the Mediterranean coast, the Middle East, and Southeast Asia. The prevalence of BD may differ among different regions, ranging from 0.12 to 370 occurrences per 100,000 individuals (Yazici et al., 1984; Dong and Shi, 1999; Sakane et al., 1999). Due to the lack of specific biomarkers, the diagnosis of BD mainly relies on variable groups of clinical manifestations, and the most widely used diagnostic criteria for BD is the International Criteria for Behçet’s Disease (ICBD) created in 2006 (Disease, International Team for the Revision of the International Criteria for Behçet’s et al., 2014). Thus, the identification of novel biomarkers to improve early and precise diagnosis of BD is needed.

The pathogenesis of BD is not well known. An individual’s genetic background, when combined with specific environmental exposure, can trigger the onset of BD. HLA-B*51 is the strongest known risk factor that was identified in 1974, and approximately 50% of BD patients carry the HLA-B*51 allele. As GWAS technology was applied as a method in BD research, more risk genes were found, including IL-10, IL-23R, IL-12RB2, IL-18, TNF-α, ERAP-1, STAT4, and IFN-γ. In addition to genetic susceptibility, environmental factors such as the presence of *Streptococcus sanguinis*, herpes simplex virus, HSP, and endothelial antigens are risk factors for BD (Gul, 2001; de Chambrun et al., 2012; Morton, Situnayake, and Wallace, 2016; Giza et al., 2018; Leccese and Alpsoy, 2019; Tong et al., 2019; Peng et al., 2020; Hirahara et al., 2022).

In addition to the presence of genetic variants and the triggers of outside infection, immunological abnormalities play an important role in the development and progression of BD (Tong et al., 2019; van der Houwen, van Hagen, and van Laar, 2022). A previous study reported that the number of NK cells in the peripheral blood of BD patients was lower than that in NCs, and this was shown to be related to disease activity (Hasan et al., 2017). Several studies have identified that the ratio of TH17/TH1 increased in BD patients because of the aberrant differentiation of naive T cells. IFN-γ and IL-12 from Th1 cells can mediate the inflammatory response of T cells and neutrophils, while IL-17 from TH17 cells can make the neutrophils hyper reactive in patients with BD (Kim et al., 2010; de Chambrun et al., 2012; Emmi et al., 2016; Nanke, Yago, and Kotake, 2017; Lucherini et al., 2018; Tong et al., 2019; van der Houwen, van Hagen, and van Laar, 2022). Furthermore, aberrantly activated neutrophils accumulate in both acute and recurrent lesions and produce ROS and NETs, which further promotes thrombosis and the inflammatory response in BD patients (Takeno et al., 1995; Sakane, Suzuki, and Nagafuchi, 1997; Yamashfta, 1997; Eksioglu-Demiralp et al., 2001; Safi et al., 2018; Le Joncour et al., 2019; Verrou et al., 2021). Monocytes are critical members of the innate immune system that bridge the innate and adaptive immune systems. The monocytes in BD patients were highly activated and could be activated by NETs that were secreted by neutrophils (Şahln et al., 1996; Neves et al., 2009; Perazzio, Andrade, and De Souza, 2020). The supernatant of monocytes isolated from the blood of BD patients could induce neutrophils to migrate into the vascular endothelium (Şahln et al., 1996). TLR2/TLR4 overexpression in the monocytes of BD patients could activate the innate immune system against external stimuli *via* the TLR pathway (Liang et al., 2013). Moreover, several studies revealed abnormal functioning of polarized macrophages with M1 macrophage-predominant inflammation and impaired M2 macrophage function in patients with BD (Nakano et al., 2018; Hirahara et al., 2022). scRNA-seq analysis of PBMCs from BD patients showed that there is a higher proportion of monocytes in BD patients than in NCs, and accumulation of C1q-high (C1qhi) monocytes contributed to the inflammation in BD patients (Zheng et al., 2022).

To date, researchers have focused on the peripheral blood of BD patients. However, there has been no high-throughput analysis of the skin tissue, which is commonly involved in BD. In this study, we performed RNA-seq analysis on the skin tissue of BD patients. Through bioinformatics analysis of the RNA-seq data, we identified a total of 260 DEGs that were mainly enriched in the regulation of immune cells, the release of proinflammatory factors, or the IFN-γ signaling pathway in BD patients compared to NCs. Furthermore, our study combined single-cell data from PBMCs and bulk RNA-seq data from skin samples to analyze the infiltrating immune cell subtypes in skin lesions. We identified a higher proportion of monocytes and a higher proportion of the ISG15 + Mono and C1q + Mono subsets in the skin when comparing BD patients to NCs.

## 2 Materials and methods

### 2.1 Patients and study design

**FIGURE 1:**
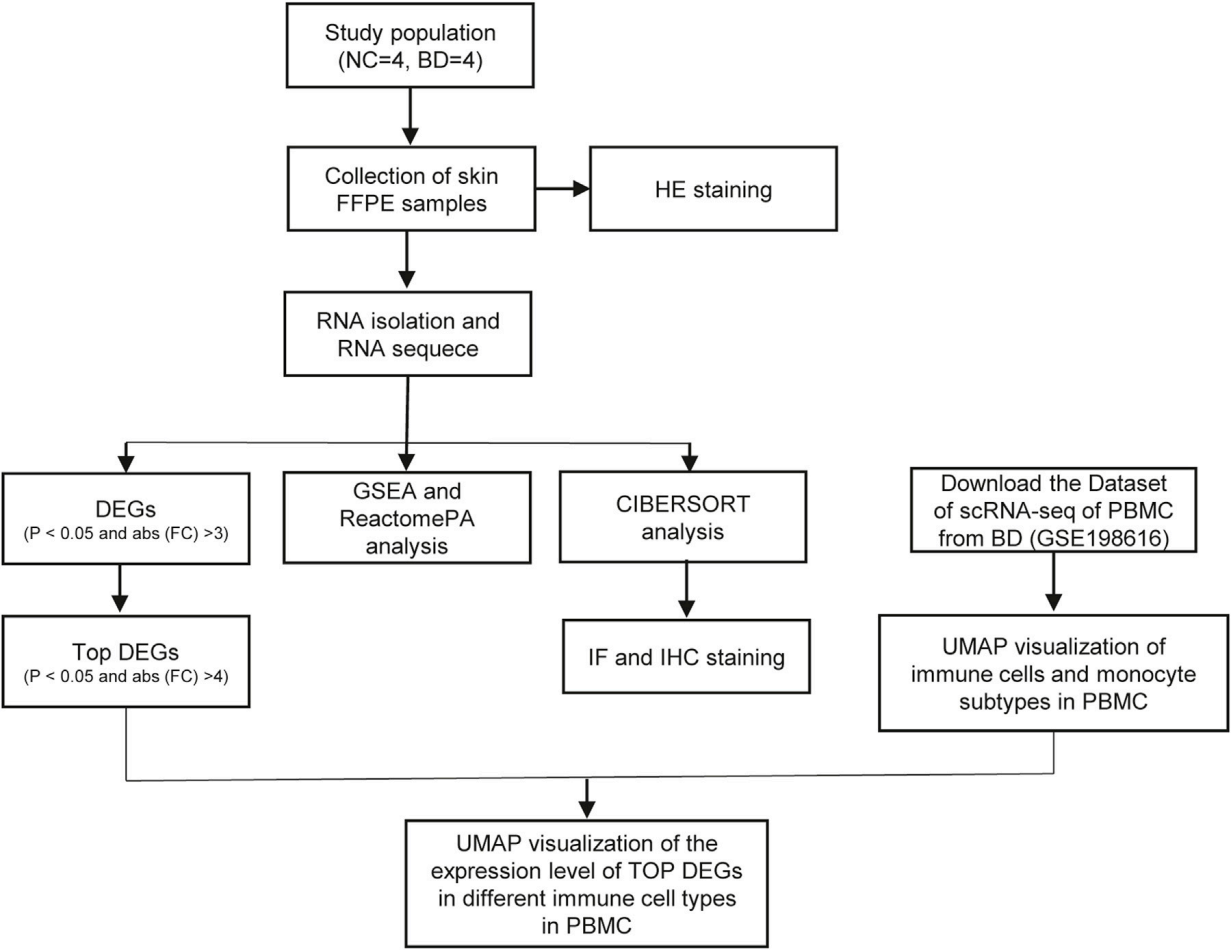
The flow chart of study design. In this study, 4 BD patients and 4 NCs were enrolled in this study. RNA-seq of the skin of BD patients and NCs were conducted. The DEGs between BD and NCs were analyzed, and functional analysis of these DEGs were conducted. Using the deconvolution method CIBERSORT, we analyzed the immune cells subtypes in skin of BD by integrating the scRNA-seq of PBMC and bulk RNA-seq data of skin. Among these immune cell types, monocyte, ISG15 + Mono and C1q + Mono were confirmed by IHC and IF staining respectively. UMAP visualized of the expression levels of top DEGs in different immune cell types in PBMC.

In this study, four patients who were diagnosed with BD based on the International Criteria for Behçet’s Disease (ICBD) were recruited from Xiangya Hospital. Skin biopsy specimens from BD patients were collected. Demographics and clinical characteristics of BD patients and NCs was shown in Table 1. The study design was shown in Figure 1.

**TABLE 1:** Demographics and Clinical characteristics of BD patients and NCs.

Characteristic	BD (n = 4)	NC (n = 4)
Age (years, mean ± SD)	29 ± 19	40 ± 13
Gender, Male (n, %)	2 (50)	3 (75)
Clinical features of BD		
ocular lesion	0 (0)	—
Genital aphthosis	1 (25)	—
Oral aphthosis	3 (75)	—
Skin lesions	4 (100)	—
Neurological manifestations	0 (0)	—
Vascular manifestations	3 (75)	—
Positive pathergy test*	0 (0)	—

### 2.2 RNA extraction, library construction and sequencing

The skin FFPE specimens were provided by the Department of Pathology in Xiangya Hospital. Total RNA was extracted using an RNeasy FFPE Kit (QIAGEN, 73504) according to the manufacturer’s protocol. RNA purity was assessed using an ND-1000 Nanodrop. Ribosomal RNA was removed using the Ribo-Zero™ kit (Epicenter, Madison, WI, United States). Then, mRNA libraries were constructed with an NEBNext^®^ UltraTM RNA Library Prep Kit for Illumina (NEB, United Ststes) according to the manufacturer’s instructions. The libraries were paired-end sequenced (PE150, sequencing reads were 150 bp) on the Illumina NovaSeq 6000 platform at Guangzhou RiboBio Co., Ltd. (Guangzhou, China).

Raw fastq sequences were analyzed with Trimmomatic tools (v 0.36). Sequencing read quality was calculated using FastQC software. Adapter removal and read trimming were performed using Trimmomatic tools. Paired-end reads were aligned to the human reference genome hg19 with HISAT2. HTSeq v0. 6.0 was subsequently employed to convert the aligned short reads into read counts for each gene model. Whole sample expression levels were presented as RPKM (expected number of reads per kilobase of transcript sequence per million base pairs sequenced).

### 2.3 Analysis of differentially expressed genes

Bioinformatics analysis with the R package edgeR was used to screen for differentially expressed genes (DEGs) in BD patients and NCs with a criteria threshold *p* < 0.05 and absolute fold change >3. Volcano plot was generated using the R package ggplot2 to show the DEGs. Then, the top DEGs were selected by the criteria *p* < 0.05 and absolute fold change >4, and shown by hierarchical clustering performed using the R package ggplot2.

### 2.4 Pathway enrichment analysis

The Gene Set Enrichment Analysis (GSEA) and ReactomePA databases were used to analyze the GO annotation and enriched pathways of the DEGs. A total of 260 DEGs, including 99 upregulated genes and 161 downregulated genes, that were identified in the skin of BD patients were used as input for analysis performed using the R package clusterProfiler. Significantly enriched pathways were determined with a cutoff of a corrected *p* < 0.05.

### 2.5 Single-cell RNA sequencing (scRNA-seq) data analysis

The scRNA-seq UMI count data from the peripheral blood mononuclear cells and monocytes of BD patients was downloaded from GSE198616. R package Seurat V4.0.0. was used for data analysis. Cells were first filtered by the number of detected UMIs per cell (nUMI; ≥1,000), the number of detected genes (nGene; ≥600), log10 Genes Per UMI (>0.80) and the ratio of mitochondrial genes observed/total number of genes observed (mitoRatio; <0.2). Sctransform was used for data normalization, and reciprocal PCA (RPCA) was used to integrate data from all of the samples (Stuart et al., 2019). Principal component analysis (PCA) and uniform manifold approximation and projection (UMAP) were used for visualization. The main clusters were first annotated by SingleR (v1.4.0) with reference datasets from HumanPrimaryCellAtlasData and BlueprintEncodeData from the Celldex package (v1.0.0) (Aran et al., 2019) and confirmed by identification of classical marker genes of immune cells. Gene expression was visualized with UMAP, which was generated by the Seurat functions Dimplot and FeaturePlot. A stacked bar chart was created by ggplot2.

### 2.6 Deconvolution analyses to detect skin-infiltrating cells in patients with BD

CIBERSORTx is an analytical tool from the Alizadeh and Newman Labs that computes gene expression profiles and provides an estimation of the abundances of specific cell types in a mixed cell population (Newman et al., 2019). The abundance of skin-infiltrating immune cells and monocytes in BD patients was quantified using the bulk RNA-seq data by CIBERSORTx. First, we used BD scRNA-seq data to generate a gene signature matrix for each cluster. Then, we utilized an S-mode batch correction and 1,000 permutations with quantile normalization disabled to analyze the relative fractions of each cell type. xCell is a webtool that performs cell type enrichment analysis from gene expression data for 64 immune and stromal cell types that was validated using extensive *in silico* simulations and cytometry immunophenotyping (Aran et al., 2017). We used xCell to analyze the RNA-seq data that were then used to describe the heterogeneous cellular expression profiles of skin from BD patients and NCs.

### 2.7 Hematoxylin–eosin (H&E) staining

Skin samples were formalin-fixed and embedded in paraffin (FFPE) at the Department of Pathology in Xiangya Hospital. The FFPE skin samples were deparaffinated and stained with hematoxylin and eosin. Then, images were acquired and analyzed using an Olympus microscope. The nuclei were stained purple, while the cytoplasmic components were stained pink.

### 2.8 IF and IHC staining

Skin FFPE tissue sections (3 μm) were deparaffinated at 70 °C in an oven for 2 h and then rinsed in xylene twice for 10 min each time. Then, slides were manually dehydrated using an ethanol series (2 × 100 ethanol, 5 min each, 2% × 95% ethanol, 5 min each, 1 × 100% ethanol, 5 min each). After washing the slides with PBS, they were subjected to heat-mediated antigen retrieval in Citrate-EDTA Antigen Retrieval Solution (Beyotime, P0086). Then, the slides were incubated in deionized water (with 3% hydrogen peroxide) for 10 min to reduce false positives in the specimens. Bovine serum albumin (5%) was used to block non-specific binding. Then, the slides were incubated overnight at 4 °C with rabbit anti-human CD14 (1:200 for IF, 1:1,000 for IHC, Proteintech-17000-1-AP), rabbit anti-human CD16 (1:500 for IHC, ab246222), mouse anti-human C1QA (1:200 for IF, Proteintech 67063-1-Ig), and rabbit anti-human ISG15 (1:100 for IF, ab285367). The corresponding secondary antibodies were added to slides and incubated for 1 h at room temperature in the dark. Antifading mounting medium (Solarbio, S2100) was then added. Images were captured using an Olympus microscope with a DP72 camera (Olympus, Shinjuku, Japan). For the homologous primary antibody, tyramide signal amplification (TSA) technology using a double-labeled multiplex immunofluorescence kit (AiFang Biological, China) was performed.

### 2.9 Statistical analysis

Results were analyzed and visualized with Prism version 9. To compare two groups, independent-sample *t*-test or paired *t*-test were used for normally distributed variables, and Wilcoxon rank-sum tests were used for non-normally distributed variables. Statistical significance was set as *p*-value <0.05.

## 3 Results

### 3.1 Differentially expressed genes (DEGs) in the skin of BD patients compared to NCs

**FIGURE 2:**
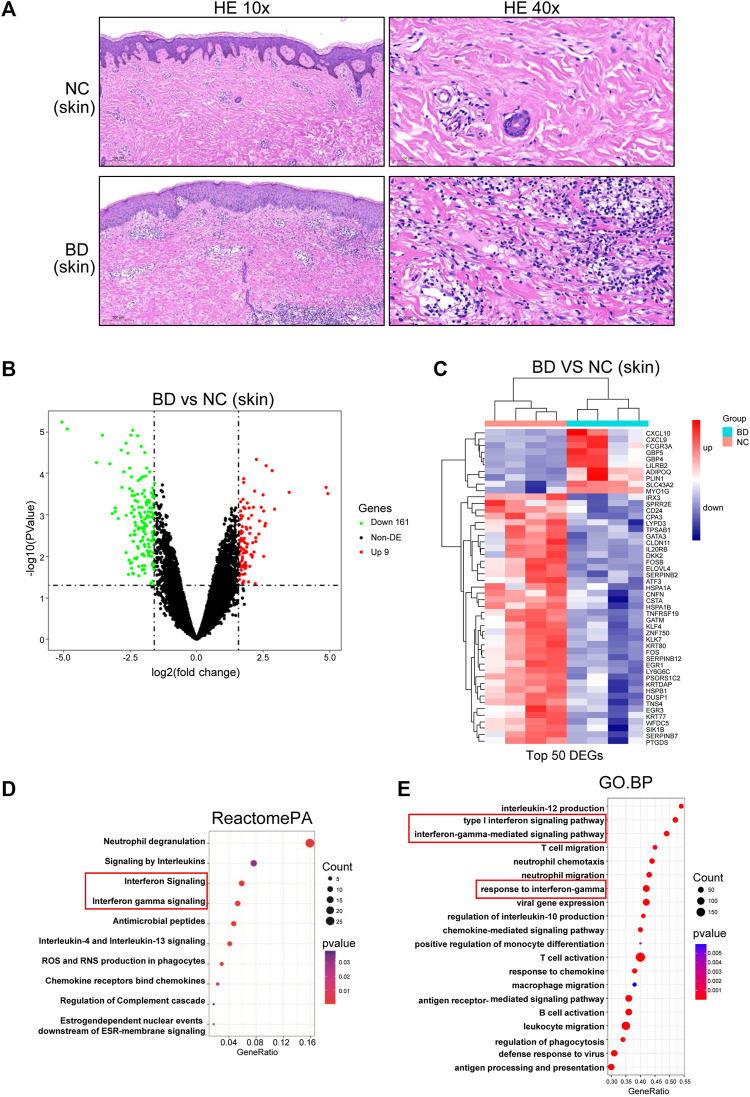
Differentially expressed genes (DEGs) in the skin of NC and BD patients. **(A)** FFPE skin samples obtained from BD patients and NCs were stained with hematoxylin and eosin (H&E). Representative images are displayed. Images in the upper panel demonstrate leukocytoclastic vasculitis in the superficial dermis and many lymphocytes and few neutrophils around the dermis and subcutaneous blood vessels. In contrast, representative images in the lower panel, which were from NCs, demonstrate that no inflammatory cells had infiltrated the skin. The scale bar of the left panel is 200 µm (magnification, ×10), and that of the right panel is 50 µm (magnification, ×40). **(B)** Volcano plot showing the DEGs in skin comparing the BD and NC samples (criteria: *p* < 0.05 and absolute fold change>3, abs (fold change) > 3). Red, significant upregulation; Green, significant downregulation; black, not significant. **(C)** Heatmap showing the top DEGs in skin when comparing BD vs. NC samples (criteria: adjusted *p* < 0.05 and abs (fold change) > 4). **(D)** ReactomePA showing the pathways enriched with the DEGs. **(E)** GSEA analysis showing the DEG enriched pathways. The depth of the color represents the adjusted *p*-value. The area of the circles in the graph indicates the gene counts.

To obtain a comprehensive understanding of the immune environment of the skin tissue, we conducted H&E staining of skin specimens from BD patients and NCs. In the skin from BD patients, there was leukocytoclastic vasculitis in the superficial dermis, and many lymphocytes and few neutrophils were found around the dermis and in subcutaneous blood vessels (Figure 2A, lower panel). In contrast, there were almost no infiltrating inflammatory cells in the NC samples (Figure 2A, upper panel). Then, we performed bulk RNA-seq analysis on skin specimens from 4 BD patients and 4 NCs. There were 99 upregulated genes and 161 downregulated genes found when comparing BD and NC skin samples with the criteria *p* < 0.05 and absolute fold change >3 (Figure 2B). We further narrowed the criteria to *p* < 0.05 and absolute fold change >4 to screen for the top DEGs. We identified CXCL10, CXCL9, FCGR3A, GBP5, GBP4, LILRB2, ADIPOQ, PLIN1, SLC43A2, and MYO1G as the top upregulated genes in BD patients compared to NCs (Figure 2C). To explore the pathways that were enriched with the DEGs, ReactomePA and GSEA analyses were conducted. We identified that the viral response pathway, the activation and migration of immune cells, the inflammatory pathway, the antigen presentation pathway, and the IFN-γ signaling pathways played crucial roles in BD (Figures 2D, E).

### 3.2 The immune cell subtypes in the skin tissue of BD patients

**FIGURE 3:**
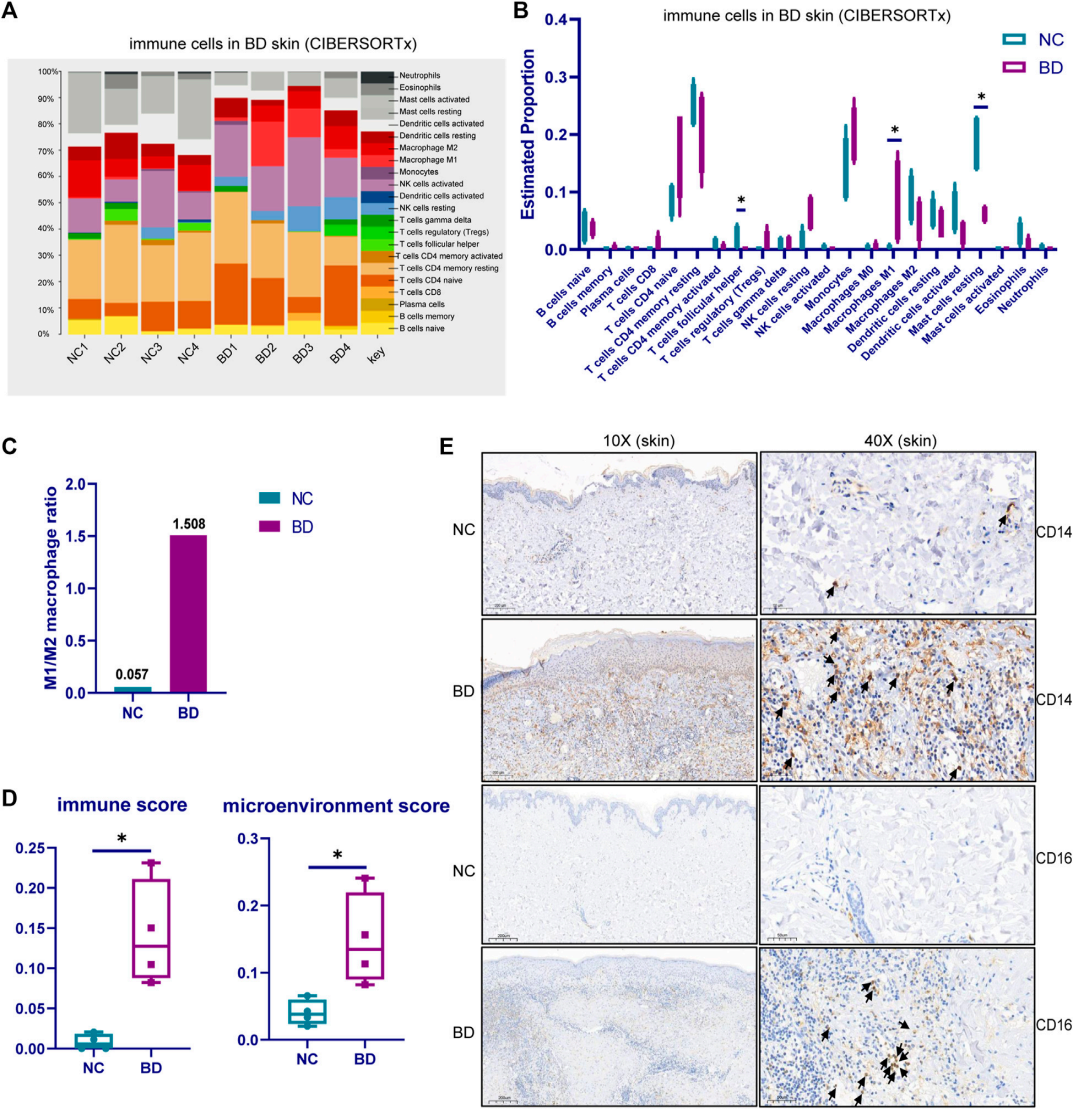
The immune cell subtypes in the skin tissue of BD. **(A)** CIBERSORTx analysis showing the cell composition of infiltrating immune cells in the skin of BD patients using bulk RNA-seq data. **(B)**Violin plot showing the cell proportion of 22 kinds of cell types in the skin of BD patients and NCs. **(C)** The ratio of M1 and M2 macrophages in BD patients compared with NCs **(D)** xCELL analysis showing the immune score and microenvironment score of the BD patients and NCs. The Wilcoxon test was applied, and a *p*-value <0.05 was considered significant. **(E)** IHC staining showing the monocyte type in the skin of BD patients and NCs.

Based on analysis of the DEGs, we knew that the activation and migration of immune cells were probably important in the pathogenesis of BD in skin lesions. Therefore, we explored the immune cell types in the skin of BD patients. First, we analyzed the scRNA-seq data from PBMCs from BD patients (GSE198616). Twenty distinctive clusters of immune cells and non-immune cells were identified, including CD8 T effector, naive B, CD4 Treg, CD8 T naive, CD4 T naive, CD4 T memory, CD8 T memory, CD14^+^ monocyte, cDC, activated NK, CD4 T IFN related, CD16^+^ monocyte, memory B, innate-like T cells, pDC, megakaryocyte, resting NK, proliferating T, and plasma B clusters (Supplementary Figure S1A). The proportion of the 20 distinctive cell types in BD and NC samples is shown by a stacked bar chart in Supplementary Figure S1B. Among these 20 cell subtypes, CD14^+^ monocytes and CD16^+^ monocytes were more abundant in BD patients than in NCs (Supplementary Figure S1C). To further investigate the proportion of immune cells in the skin of BD patients, we deconvoluted the immune cell subtypes using bulk RNA-seq data by CIBERSORT with the LM22 signature matrix (Figure 3A, B). For the high proportion of monocytes in PBMC of BD and the proinflammatory function of monocytes in BD (Zheng et al., 2022). We focused on monocyte subtypes in skin of BD. The number of monocytes increased in skin which was validated by IHC staining using CD14 and CD16 marker (Figure 3E). Furthermore, the M1/M2 macrophage ratio in BD patients was higher than the ratio in NCs (Figure 3C). Macrophage M1 has proinflammatory characteristics that could secrete IL-6 and TNF-alpha to induce inflammatory reactions, while macrophage M2 has anti-inflammatory characteristics; the reduced level of macrophage M2 in BD patients could further promote the inflammatory reactions (Peng et al., 2020). The xCell analysis was used to estimate the immune score and microenvironment score of BD patients and NCs (Figure 3D). Both the immune score and microenvironment score were higher in BD patients than in NC patients, which illustrated that more immune cells accumulated in the skin lesions of BD patients. Taken together, these results showed that there were more monocytes in the skin of BD patients, which likely induced the inflammatory response.

### 3.3 Heterogeneity of monocyte subtypes in the skin tissue of BD patients

**FIGURE 4:**
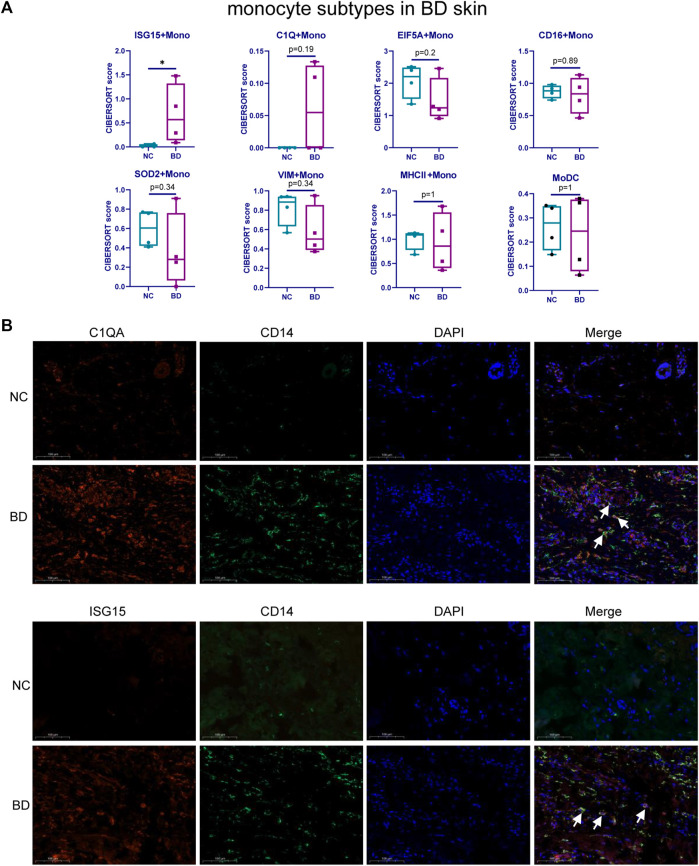
Heterogeneity of monocyte subtypes in BD patients. **(A)** Boxplot comparing the relative proportion of monocyte subsets in skin tissue of BD patients and NCs analyzed by CIBERSORTx. The Wilcoxon test was applied. **(B)** IF staining of CD14+C1QA+ and CD14+ISG15+ in the skin of BD patients and NCs. Representative images show CD14+C1QA+ and CD14+ISG15 + positive cells in the skin of BD patients and NCs. CD14 (green), C1QA (red), ISG15 (red).

Monocytes are highly heterogeneous and are generated through distinct developmental pathways. Thus, they have a diverse set of functions (Guilliams, Mildner, and Yona, 2018). To identify the subsets of monocytes that are found at higher proportions in BD patients, we assessed the heterogeneity of monocytes in PBMCs and infiltrated skin from BD patients. We used UMAP to analyze the scRNA-seq data from isolated CD14^+^ monocytes from BD patients and NCs (GSE198616). These results allowed us to visualize the subtypes of monocytes in PBMCs and eight distinctive clusters, including ISG15 + Mono, C1Q + Mono, EIF5A + Mono, CD16+Mono, SOD2+Mono, MHCⅡ+Mono, VIM + Mono and MoDC, which were shown in Supplementary Figure S2A. The stacked bar chart shows the relative proportion of eight subsets of monocytes in PBMCs from BD patients or NCs (Supplementary Figure S2B). Then, we deconvoluted the eight subsets of infiltrating monocytes in the skin of BD patients and NCs by CIBERSORT (Figure 4A). It turns out that there is a higher proportion of the ISG15 + Mono and C1Q + Mono subsets in the skin of BD patients than in NCs. We further conducted IF staining of CD14, C1QA and ISG15 in skin specimens from BD patients and NCs (Figure 4B). The results showed that the number of CD14^+^ C1QA^+^ positive and CD14^+^ ISG15^+^ positive cells increased in the skin of BD patients.

### 3.4 Expression of the top upregulated DEGs in the immune cell subtypes found in the PBMCs of BD patients and NCs

**FIGURE 5:**
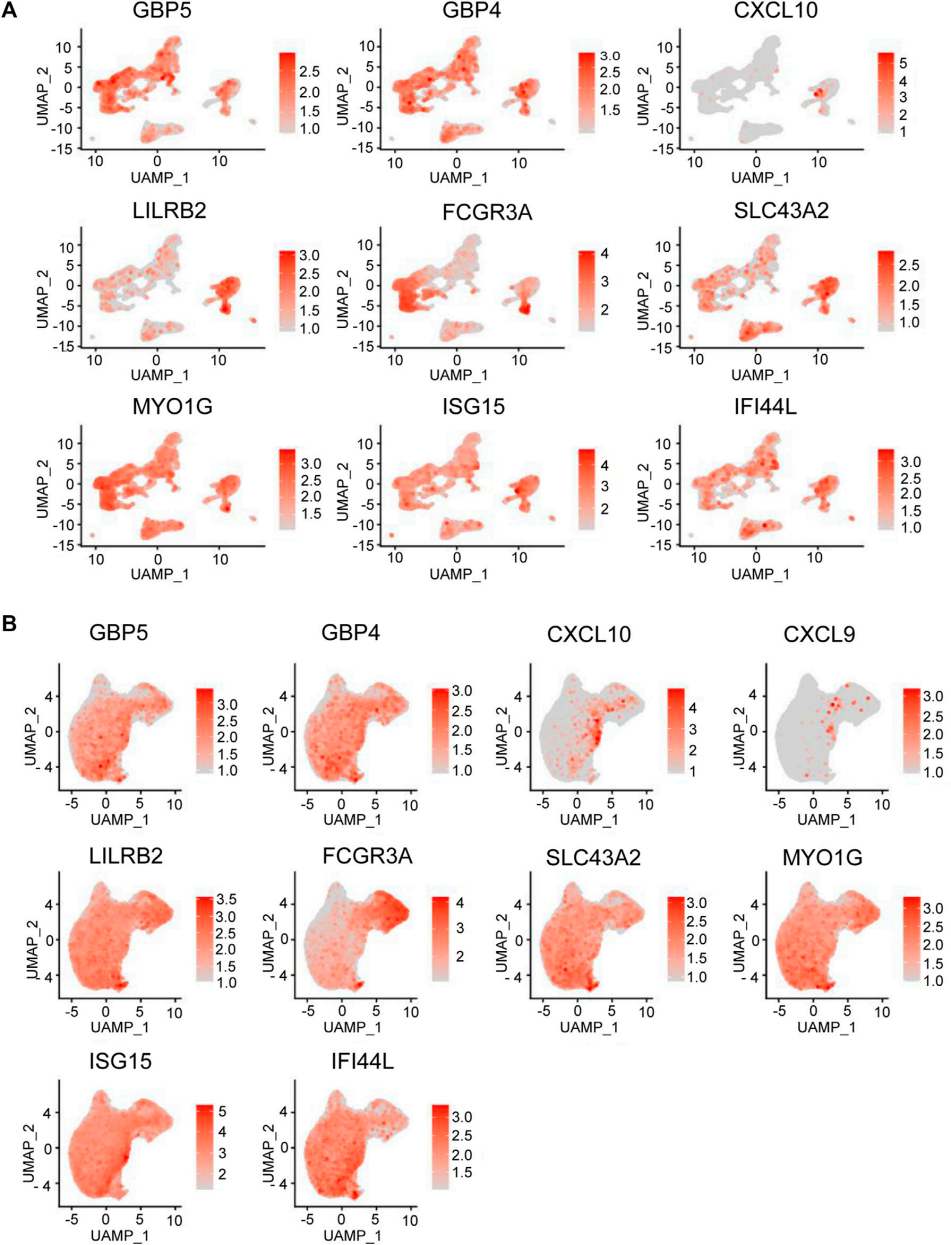
DEG expression in BD immune cell subtypes. **(A)** Feature plot representing the average expression level of the genes in different cell types that are highly upregulated in PBMCs from BD patients. **(B)** Feature plot representing the average expression level of the genes in different monocyte cell types that are highly upregulated in PMBCs from BD skin.

To better understand the mechanism of aberrant immunity in BD patients, we explored the average expression level of top upregulated genes in different cell types of PBMCs. (. We could see that GBP5, GBP4, CXCL10, LILRB2, FCGR3A, SLC43A2, MYO1G, ISG15, and IFI44 L were all expressed in monocyte type (Figure 5A). Then, we analyzed the average expression level of these genes in monocyte subtypes. CXCL10 was specifically expressed in ISG15 + monocyte, and GBP5, GBP4 and IFI44L were expressed more strongly in ISG15 + monocytes. LILRB2 was expressed more strongly in CD16^+^ monocytes and C1Q + monocytes (Figure 5B). Altogether, The result showed that top upregulated genes identified in skin expressed in monocyte type from PBMC and some genes expressed specifically or strongly in the ISG15+ and C1q + monocyte subtype.

## 4 Discussion

In this study, we conducted bulk RNA-seq analysis of skin tissues from BD patients. We identified CXCL10, CXCL9, FCGR3A, GBP5, GBP4, LILRB2, ADIPOQ, PLIN1, SLC43A2, and MYO1G as the top upregulated genes in BD patients compared to NCs. CXCL10 and CXCL9 are ligands for the chemokine receptor CXCR3 and are mainly secreted by monocytes, T cells, and NK cells. It has been reported that CXCL10 and the CXCL9/CXCR3 axis can regulate immune cell migration, differentiation, and activation (Tokunaga et al., 2018). GBP4 and GBP5 are members of the IFN-induced GTPase family and localize in a single cluster on chromosome 1. Previous studies have shown that induction of the GBPs, including GBP4 and GBP5, was the key to triggering antimicrobial activity against intracellular bacteria (MacMicking, 2004; Taylor, Feng, and Sher, 2004; Kim et al., 2012). FCGR3A, also named CD16, was linked to patient susceptibility to recurrent viral infections (Vivier et al., 2008), and its gene polymorphisms were related to patient susceptibility to BD (Zhang et al., 2018). LILRB2 is a member of the leukocyte immunoglobulin-like receptor (LIR) family and is expressed selectively in monocytes, macrophages, and dendritic cells (Colonna et al., 1998). LILRB2 was thought to control inflammatory responses, control cytotoxicity, help focus the immune response and limit autoreactivity (Brown, Trowsdale, and Allen, 2004). Moreover, it was found that LILRB2 could inhibit macrophage functions (Chen et al., 2018). Interestingly, we found that CXCL9, CXCL10, GBP5, and GBP4 could all be induced by IFN-γ, and the genes that were differentially expressed in the skin of BD patients and NCs were significantly enriched in the IFN-γ signaling pathway. Furthermore, several studies have verified that IFN-γ levels are significantly increased in BD patients (Bacon et al., 1984; MacMicking, 2004; Taylor, Feng, and Sher, 2004; Kim et al., 2012; Tokunaga et al., 2018; Talaat et al., 2019). This strongly suggests that the IFN-γ signaling pathway probably plays a crucial role in the pathogenesis of BD.

Monocytes are highly heterogeneous and have a variety of functions (Guilliams, Mildner, and Yona, 2018). A previous study reported that the population of IM (CD14++CD16^+^) cells was higher in PBMCs from BD patients than in those from NCs, and the population of NCM (CD14^+^CD16++) cells was lower in these patients; furthermore, the populations of both cells were related to the ESR and CRP levels (Li C et al., 2021). Another study revealed that the C1q-high monocyte subtype was increased in the blood of BD patients and this subtype had a proinflammatory effect through the IFN-γ signaling pathway in BD patients (Zheng et al., 2022). In addition to BD, monocytes can infiltrate the tissues of patients with several other autoimmune diseases, such as SLE, RA, SSC, and SS, and they can secrete several cytokines and chemokines to stimulate and recruit other immune cells to disease tissue (Janossy et al., 1981; Ishikawa and Ishikawa, 1992; Sack, Stiehl, and Geiler, 1994; Carlsen et al., 2004; Bramow et al., 2010; Misharin et al., 2014). In our study, we found that more monocytes infiltrated the skin of BD patients than the skin of NCs, and there was a higher proportion of the C1q + monocyte and ISG15 + monocyte subsets. This likely suggests that specific monocyte subtypes play a major role in the development of BD.

The differentiation of monocytes varies in different autoimmune diseases and is affected by the local environment and the disease stage (Piccolo et al., 2017; Ma et al., 2019). Previous studies revealed that there was an increased amount of M1 macrophages, which can secrete IL-6 and TNF-alpha to induce an inflammatory reaction, in the blood of BD patients, while the amount of macrophage M2, which has anti-inflammatory characteristics, was reduced in the blood of BD patients, further inducing the inflammatory reaction in these patients (Nakano et al., 2018; Shi et al., 2019; Hirahara et al., 2022). In our study, we identified that the aberrant differentiation of monocytes in the skin of BD patients favored differentiation into M1 macrophages rather than M2 macrophages. Moreover, the main characteristic of the skin of BD patients was the overactivation of neutrophils in the lesion site; in addition, NETS secreted by neutrophils could activate and mediate the monocytes on the skin (Safi et al., 2018; Li L et al., 2021). This phenomenon explains why there is a high abundance of monocytes in patients with BD. and suggests that monocytes likely connect both the innate and adaptive immune systems to induce the development of BD.

The activation, differentiation, and migration ability of immune cells are regulated by specific genes. CXCL10 was expressed mainly in CD14^+^ monocytes. LILRB2 was expressed strongly in CD14^+^ monocytes and CD16^+^ monocytes. FCGR3A was expressed strongly in CD8 T effector cells, activated NK cells, and CD16^+^ monocytes. We identified that GBP5, GBP4, LILRB2, SLC43A2, MYO1G, ISG15, and IFI44 L were expressed in almost all eight monocyte subtypes. CXCL10 was expressed mainly in the ISG15 + Mono subset, and FCGR3A (CD16) was expressed strongly in the C1Q + Mono and CD16+Mono subsets. Altogether, these results showed that different genes were expressed in specific cell types, and the genes that are expressed are likely correlated with the mechanism of the migration and activation of the specific cell types. Further research should be conducted to verify how these genes regulate the function of specific immune cells in BD patients.

There were some limitations in our study. First, because of the low frequency of BD, the sample size was small, which may decrease the statistical power. Second, the clinical relevance of the identified DEGs and cell types, especially monocyte subsets, in BD patients was not investigated. In the future, we could explore the correlation between the DEGs and the infiltration ability of cells in the skin. We could also explore the detailed contribution of these cells to the development of skin lesions in patients with BD.

The characteristic recurrent symptoms of BD place a large amount of economic and emotional pressure on patients. Skin is the most basic tissue and the primary tissue involved in BD. Because BD is a chronic inflammatory vasculitis, studies on BD have focused on PBMCs. In this study, we combined single-cell sequencing data (GSE198616) and RNA-seq data of skin from patients with BD to explore the potential causative genes, pathways, and predominant infiltrating cells responsible for inducing BD.

## Data Availability

The data presented in the study are deposited in the SRA repository, accession number SRR22877276 (Available at: https://www.ncbi.nlm.nih.gov/sra/?term=SRR22877276).

## Supplementary Material

Supplementary Figure S1 Immune cell types from PBMC of BD patients and NCs by analyzing scRNA-seq data. **(A)** UMAP visualization of different cell clusters in PBMCs by scRNA-seq data (GSE198616), colored by the identified subtypes. **(B)** Stacked bar chart showing predicted cell fractions in PBMCs from BD patients and NCs.

Supplementary Figure S2 Monocyte subtypes from PBMC of BD patients and NCs by analyzing scRNA-seq data of CD14^+^ monocytes. **(A)** UMAP plot representing the monocyte subtypes in PBMCs using scRNA-seq data of isolated CD14^+^ monocytes (GSE198616), colored by the identified monocyte subtypes. **(B)** Stacked bar chart summarizing the relative proportion of monocyte subtypes in PBMCs from BD patients and NCs.
