# Supplementary material for: The proportion of C1q-high and ISG15-high monocytes in the skin of patients with Behçet disease
Source: Front Pharmacol. 2023 Mar 7;14:1110741. doi: 10.3389/fphar.2023.1110741 (PMC10028182; doi:10.3389/fphar.2023.1110741)
Supplement: Supplementary file 1 [file DataSheet1.pdf]

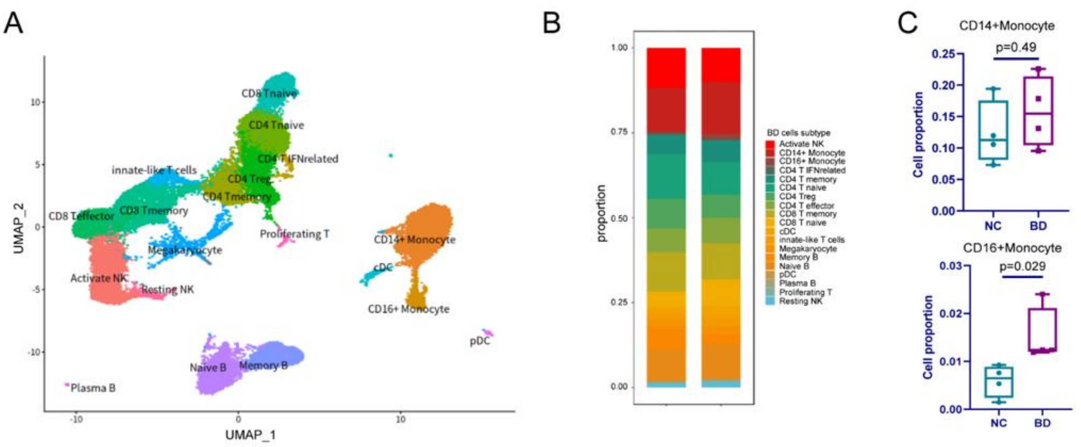

Supplementary Figure1. (A) UMAP visualization of different cell clusters in PBMCs by scRNA-seq data (GSE198616), colored by the identified subtypes. (B) Stacked bar chart showing predicted cell fractions in PBMCs from BD patients and NCs.

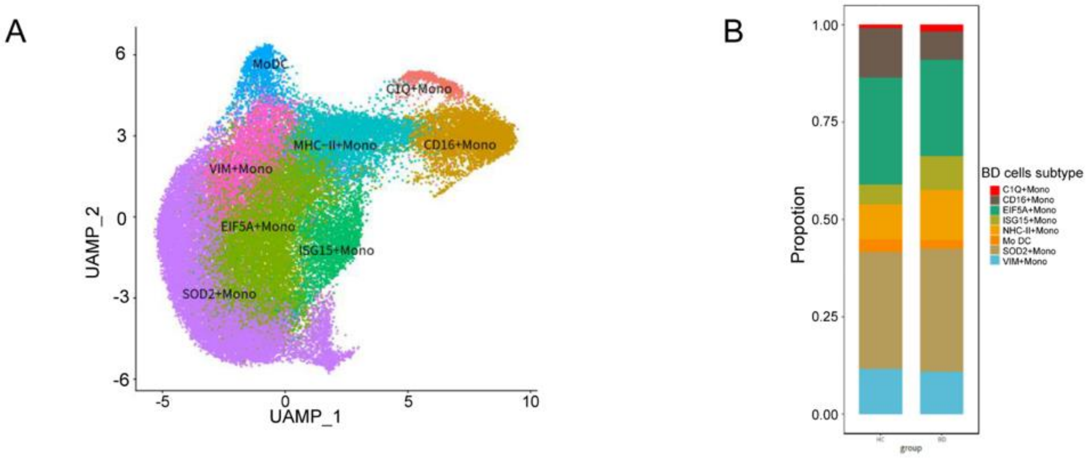

Supplementary Figure 2. (A)UMAP plot representing the monocyte subtypes in PBMCs using scRNA-seq data of isolated CD14+ monocytes (GSE198616), colored by the identified monocyte subtypes. (B) Stacked bar chart summarizing the relative proportion of monocyte subtypes in PBMCs from BD patients and NCs
